# Neuroticism developmental courses - implications for depression, anxiety and everyday emotional experience; a prospective study from adolescence to young adulthood

**DOI:** 10.1186/s12888-014-0210-2

**Published:** 2014-08-06

**Authors:** Maren Aldinger, Malte Stopsack, Ines Ulrich, Katja Appel, Eva Reinelt, Sebastian Wolff, Hans Jörgen Grabe, Simone Lang, Sven Barnow

**Affiliations:** Department for Clinical Psychology and Psychotherapy, Ruprecht-Karls-University, Hauptstraße 47-51, Heidelberg, 69117 Germany; University Medicine Greifswald, Ellernholzstraße 1-2, Greifswald, 17475 Germany; HELIOS Hospital, Große Parower Straße 47-53, Stralsund, 18435 Germany

**Keywords:** Neuroticism, Anxiety, Depression, Ecological momentary assessment, Emotional experience

## Abstract

**Background:**

Neuroticism is frequently discussed as a risk factor for psychopathology. According to the maturity principle, neuroticism decreases over the course of life, but not uniformly across individuals. However, the implications of differences in personality maturation on mental health have not been well studied so far. Hence, we hypothesized that different forms of neuroticism development from adolescence to young adulthood are associated with differences in depression, anxiety and everyday emotional experience at the age of 25.

**Methods:**

A sample of 266 adolescents from the general population was examined three times over ten years (age at T_0_: 15, T_1_: 20 and T_2_: 25) using questionnaires, interviews and ecological momentary assessment (EMA). At all measurement points, neuroticism was assessed with the NEO inventory. At T_2_, diagnoses of major depression and anxiety disorders were captured with a structured clinical interview (M-CIDI). Phone-based EMA was used to assess emotional experience and affective instability over a two-week period at T_2_.

**Results:**

The best fitting model was a latent class growth analysis with two groups of neuroticism development. Most individuals (n = 205) showed moderate values whereas 61 participants were clustered into a group with elevated neuroticism levels. In both groups neuroticism significantly changed during the ten year period with a peak at the age of 20. Individuals with a higher absolute level were at 14-fold increased risk for depression and 7-fold risk for anxiety disorders at the age of 25. In EMA, increased negative affect and arousal as well as decreased positive emotions were found in this high group.

**Conclusions:**

Other than expected, personality did not mature in our sample. However, there was a significant change of neuroticism values from adolescence to young adulthood. Further, over 20% of our participants showed a neuroticism development which was associated with adverse outcomes such as negatively toned emotional experience and a heightened risk to suffer from depressive and anxiety disorders in young adulthood. These high-risk persons need to be identified early to provide interventions supporting continuous personality maturation.

**Electronic supplementary material:**

The online version of this article (doi:10.1186/s12888-014-0210-2) contains supplementary material, which is available to authorized users.

## Background

Personality traits are frequently discussed as risk factors for various psychopathological complaints [1],[2]. Especially, neuroticism is often examined in the context of psychopathology [3],[4]. Individuals scoring high on this personality dimension can be characterized as worried, emotionally unstable, overly reactive or nervous [5]. Particularly, the association between high neuroticism and internalizing disorders like depression [6],[7] or anxiety disorders [8],[9] is well established.

Originally, personality traits were described to reflect genetically determined and relatively stable interindividual differences e.g., [10],[11]. However, by now there is increasing evidence that personality changes in all periods of life (for reviews see [12],[13]) with great developmental steps during adolescence/young adulthood and again in old age [14]-[16]. For instance, Littlefield, Sher, and Wood [17] found mean-level decreases of neuroticism in young adulthood. Similar results were obtained by Specht, Egloff, and Schmukle [16]. They reported that emotional stability, which is often used as synonym for low neuroticism, rises during a four year interval in different age groups in the general population. Such developmental changes of personality are summarized in the *maturity principle*[13],[18],[19]. This principle states that in most people personality matures over time. Regarding neuroticism, a decrease of neuroticism is expected. Importantly, Caspi et al. [13] emphasize that this maturation process is not uniform across individuals. Instead the authors argue that not all individuals achieve a mature personality or at least not at the same time. This should in turn be associated with differences in outcomes such as mental health or well-being, for example [13].

Thus, the maturity principle emphasizes the possibility that individuals do not mature at the same pace. Therefore, individual courses of personality maturation and their impact on mental health need to be focused on [20]. Nevertheless, only few studies examined personality changes by differentiating developmental courses (e.g., [21],[22]). For instance, three developmental groups were identified by Robins, Fraley, Roberts, and Trzesniewski [23]: in 23% of participants neuroticism levels decreased over a 4-year-interval, in 4% they increased and in 73% neuroticism remained stable. However, in this study the implications of group membership on possible outcomes like psychopathology or well-being were not examined.

Johnson, Hicks, McGue, and Iacono [24] focused on the temperament factor harm avoidance (HA), which is strongly associated with neuroticism [25], in a female twin sample. In their study, they found four different forms of development from age 14 to age 24: Three groups showed increasing HA-values, but differed in absolute level, whereas in the fourth group a decrease of HA was found. In addition, these developmental groups differed significantly regarding the prevalence of antisocial behaviour or substance dependence at the age of 24. Mroczek and Spiro [26] even found different mortality rates dependent on neuroticism level and course in middle-aged to old men. Men who scored high on this trait at baseline and who increased over a period of 18 years were less likely to survive.

Of course, this review of studies is not exhaustive. Nevertheless it gives first evidence for the serious consequences of missed personality maturation, albeit studies longitudinally relating changes in neuroticism to psychopathology are still rare. It must be noted, that, except for Johnson et al. [24] all above mentioned studies assessed psychopathology on a subsyndromal level using self-reports. In contrast, the influence of personality maturation on diagnoses of psychiatric disorders, as measurable with structured clinical interviews, has been neglected so far.

In addition, self-reports often generate global indices of impairments in general and are prone to retrospective bias [27]. It remains unclear how different forms of development influence everyday life. Such shortcomings could be overcome by ecological momentary assessment (EMA); [28]. This approach allows capturing real-time information while individuals go about their normal lives. As alterations in affectivity can be found in most mental disorders [29], the assessment of emotional experience using EMA could provide valuable additional information. With this method, emotions can be recorded in the moment they are experienced without being subject to recollection bias or other systematic distortions [30]. Further, affect dynamics such as instability or variability can be examined aside from mean levels [31],[32]. Due to its high ecological validity and enhanced flexibility compared to traditional assessment methods, EMA has gained increased application in the context of mood and affective components of mental disorders [33],[34].

When it comes to neuroticism and emotional experience, EMA is also increasing in importance [35]-[40]. For instance, Miller, Vachon, and Lynam [41] contacted undergraduate students via palm computers eight times a day over one week. In doing so, they found questionnaire-based neuroticism to be positively associated with mean negative affect and negative affect instability in daily measures. Similar results were obtained in other studies in which the authors reported more frequent, more intense and longer lasting unpleasant affect in EMA in association with neuroticism [42]-[44]. In several studies, Suls et al. [45] found that individuals with high neuroticism values strongly respond to daily problems – a pattern which they call the *neurotic cascade*. Further, in one study that assessed neuroticism at multiple measurement points, these values were aggregated over time for further analyses [46]. The authors reported lower positive affect and increased negative affect variability in individuals with high neuroticism values. To sum up, EMA methods are applied more and more frequently in research on the association between neuroticism and affect in everyday life. However, none of the above mentioned studies examined the longitudinal relationship between neuroticism and affectivity in everyday life in a representative sample taking changes in personality into account.

In line with the existing literature, we hypothesized that neuroticism values change from adolescence to young adulthood. In particular, we assumed that these changes in neuroticism are not uniform across individuals. Instead, groups of different courses should be identifiable. As all but one study examined outcomes of personality maturation using self-reports, in our study we aimed at describing the implications of group membership more precisely. Therefore, a multimethod approach was chosen: first, we examined whether belonging to a specific developmental group is associated with different degrees of psychopathology assessed by structured clinical interviews and self-ratings. Further, we tested the influences of group membership on emotions in everyday life using EMA.

## Methods

### Participants

The sample was drawn from the population-based Greifswald family study [47],[48], a subpopulation of the Study of Health in Pomerania, Germany (SHIP; John et al., [49]). In SHIP, 4308 people aged 20 to 79 were randomly selected between March 1997 and May 2000, proportional to the population size of each community, and stratified by age and gender. From this sample, 527 families who lived in a household with at least one offspring between the ages of 11 and 18 years were invited to take part in the family study. 141 families could not be located or did not answer our phone calls and letters. Further, 71 families refused to participate, resulting in a final sample of 315 families with whom assessments of parents and offspring (n = 381, mean age 15.1, SD = 2.3) were conducted (T_0_).

Parents and offspring were again investigated about five years later between 2005 and 2008 (T_1_): 87.7% of offspring (n = 334, mean age 19.6, SD = 2.4) took part in this follow-up. Since May 2011 offspring were examined for a third time (T_2_). Data of this second follow-up are available from 85.0% (n = 284) of T_1_ participants. 23 former participants were not available via post sendings or telephone calls because they moved away. 25 individuals were contacted but refused to participate and two persons died between T_1_ and T_2_. Individuals who took part in all assessments did not differ from those who dropped out after T_0_ regarding sex (*χ*^2^ = 2.37, p = .146), age (F = 2.05, p = .153), neuroticism (F = 0.73, p = .395; operationalized as harm avoidance in children younger than 16 years: F = 0.10, p = .747) and psychopathology (F = 0.16, p = .690) at T_0._ In 18 participants, at least one relevant questionnaire or interview was missing completely. These individuals were excluded from our analyses, resulting in a final sample of 266 young adults (56.4% female, mean age 24.9, SD = 2.3). Written informed consent was obtained from all participants after the study has been fully explained. The study was approved by the local ethics committee of the Ruprecht-Karls-University Heidelberg, Germany.

### Materials and procedure

An overview over all constructs and their assessment at each measurement point can be seen in Table 1.

**Table 1:** **Constructs, measures and measurement mode for the three measurement points**

	Measure	Mode	Transformation
**Neuroticism**			
T_0_			
• age < 16	harm avoidance scale of the J-TCI	self-report	scale 1 to 5
• age ≥ 16	NEO-FFI	self-report	-
T_1_	NEO-PI-R (only corresponding FFI-Items)	self-report	-
T_2_	NEO-FFI	self-report	-
**Subsyndromal psychopathology**			
T_0_	YSR total score	self-report	scale 1 to 5
T_1_	SCL-90-R: GSI	self-report	scale 1 to 5
T_2_	BSI: GSI	self-report	scale 1 to 5
**Diagnoses of depression and anxiety disorders**			
T_0 lifetime_			
• age < 16	children version of the DIPS	structured clinical interview	
• age ≥ 16	DIA-X	standardized clinical interview	
T_1__lifetime_	DIA-X	standardized clinical interview	
T_2 current_ & _lifetime_	DIA-X	standardized clinical interview	
**Emotions in everyday life**			
T_2_	ecological momentary assessment	phone-based self-report	

*Notes.* J-TCI: Junior Temperament and Character Inventory; NEO-FFI: NEO Five Factor Inventory; NEO-PI-R: NEO Personality Inventory Revised; YSR: Youth Self Report; SCL-90-R: Symptom Checklist 90 Revised; GSI: Global Severity Index; BSI: Brief Symptom Inventory; DIPS: Diagnostic Interview for Mental Disorders.

### Assessment of neuroticism

At all points of measurement, neuroticism was assessed with versions of the NEO personality inventory [49]. The NEO measures the Big-Five personality traits extraversion, neuroticism, openness, agreeableness and conscientiousness on a 5-point likert-type scale. The versions solely differ in their item number: whereas the NEO-Five-Factor-Inventory (T_0_ & T_2_; NEO-FFI; [50]) consists of 60 items, the NEO-Personality-Inventory-Revised (T_1_; NEO-PI-R; [51]) has 240 items. We only included the corresponding NEO-FFI-items from the NEO-PI-R in our analyses. Validity [52] and reliability of the NEO-FFI were found to be satisfying (Cronbach’s α T_0_: 0.716, T_1_: 0.870, T_2_: 0.868).

As the NEO is not applicable in children younger than 16 years [53], we used the harm avoidance subscale of the Junior Temperament and Character Inventory J-TCI; [54] in younger participants at T_0_ instead. This is an adapted version of Cloninger’s Temperament and Character Inventory (TCI); [55]. The harm avoidance scale comprises the subscales anticipatory worry, fear of uncertainty, shyness, and fatigability. Cronbach’s α in our sample was 0.775. Studies examining personality with multiple questionnaires found harm avoidance and neuroticism to be highly correlated and to compose a common dimension in factor analysis [56],[57]. Thus, Aluja and Blanch [25] concluded that both scales measure equivalent constructs. In our study, individuals older than 16 years answered both the NEO and the TCI at T_0_ and harm avoidance and neuroticism were highly correlated (r = .614, p = .000). To enhance comparability between measurements, we transformed the J-TCI harm avoidance scale into the NEO 1 to 5 answering mode.

### Assessment of psychopathology

#### Diagnoses of depression and anxiety disorders

At all measurement points, diagnoses of depressive and anxiety disorders were assessed with the standardized Munich-Composite International Diagnostic Interview (DIA-X/M-CIDI); [58] in individuals older than 15 years. All interviews were conducted by trained clinical psychologists either in person or via telephone if a participant was living too far away. Unfortunately, we were not able to tape our interviews. Hence, inter-rater-reliability of our diagnostic interviews could not be calculated. However, according to the developers of the DIA-X, inter-rater reliability of this interview is high (κ = .81 - 1.0) and validity according to comparison with clinical diagnoses is at least satisfying (κ = .39 - .82) [59]. As the DIA-X is not applicable in children younger than 16, at T_0_ the child version of the Diagnostic Interview for Mental Disorders (DIPS); [60] was used. The DIPS is a structured clinical interview with satisfying to good psychometric properties [60].

#### General psychopathological complaints

At first assessment, the German version of the Youth Self Report (YSR); [61],[62] was used for examination of general psychopathological complaints. The YSR is a self-report instrument and consists of 112 items assessing behavioural and emotional problems on eight scales in adolescents aged 11 to 18. A general psychopathology score was calculated from 101 items. In our study, reliability was excellent (Cronbach’s α = 0.921).

At T_1_, general psychopathology was measured with the German version of the Symptom Checklist-Revised (SCL-90-R); [63],[64] and at T_2_ with its short form, the Brief Symptom Inventory (BSI); [65], respectively. Both are self-rating inventories with nine scales assessing different symptoms during the last seven days and were found to be comparable [66]. Reliability and validity were found to be excellent for both, the SCL-90-R and the BSI (T_1_ SCL-90-R: Cronbach’s α = 0.965; T_2_ BSI: Cronbach’s α = 0.963) [67],[68]. To assess general psychopathology, the Global Severity Index (GSI); [63] was calculated for both questionnaires. In order to enhance comparability with neuroticism and interpretability of these different measurements, all instruments were transformed into a 1 to 5 response format.

### Emotional experience in everyday life

An ecological momentary approach was used to gather information regarding emotional experience in everyday life at T_2_. Computer-based phone calls were made with the SmartQ/DialQ software package (© Telesage Inc.), and recorded questions were red out by a staff member. Participants were called on their cell phones three times a day, every second day during a two week period. If the call was not answered, two additional trials were made 30, respectively 60, minutes later. Besides other questions, we asked the participants how they felt in the current moment. First, the emotional state was examined in general by indicating current valence (from good to bad) and arousal (from relaxed to tense). Answers were given on likert-type scales ranging from 0 to 6 by pressing the according number on the keyboard. Second, we asked more specifically for the experience of eight different emotions (happiness, sadness, disgust, anxiety, anger, interest, shame, boredom) again using scales from 0 to 6. Higher values indicated stronger momentary experience of this particular emotion. To date, methodology in EMA studies is manifold, and standardized questions and instruments are missing so far [33]. Nevertheless, as mentioned above, these designs are meant to diminish recall biases and increase ecological validity compared to self-report questionnaires. Further, there is some literature reporting good reliability and validity of EMA in clinical psychology research [69],[70].

### Data analyses

**Figure 1:**
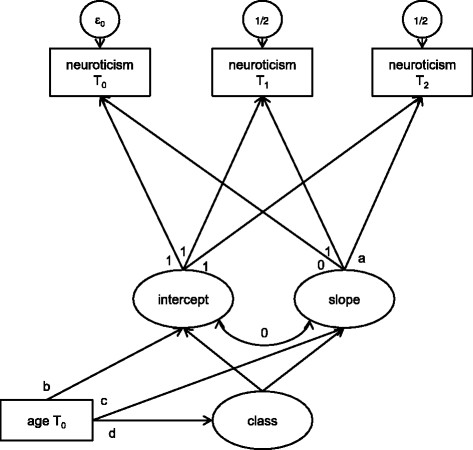
**Latent class growth analysis model for neuroticism at three measurements points.** Legend: estimated path coefficients for a 2-class-solution: a = 0.655, p ≤ .001; b = -0.001, p = .972; c = -0.063, p = .003; d_(N__moderate)_ = -0.233, p = .049; d_(N high)_ = 0.233, p = .049.

When it comes to modelling longitudinal growth data, various approaches can be used [71]. In this study we examined two different models, namely latent class growth analysis (LCGA) and growth mixture modelling (GMM). Both models were conducted with neuroticism at the three measurement points using M*plus* version 6 [72]. As an extension to conventional latent growth models, LCGA and GMM allow to identify latent groups with different developmental trajectories. Individuals are grouped based on latent growth factors, namely intercept (initial status) and slope. In our models the factor loading for the slope growth factor on T_2_ was freely estimated. Due to our relatively large age range as well as differences in neuroticism assessment depending on age at T_0_, age was included as a covariate. Further, the error variances of T_1_ and T_2_ neuroticism were set to be equal because at these assessments the same instrument was used as opposed to T_0_. LCGA is a specific form of GMM in which trajectories within a class are defined to be homogenous, i.e., the variance of the slope factor is fixed to zero within groups (see Figure 1). In contrast, in GMM the variance of the slope factor is freely estimated. Thus, the slope factor can covariate with other variables such as the intercept, (for example for a detailed description of LCGA and GMM see [73]). In M*plus*, a variety of indices is provided to evaluate model fit. In this study, the best group solution was identified on the basis of the following criteria [74]: the Bayesian information criterion (BIC, lowest values considered best), the Lo-Mendell-Rubin-test (LMR); [75] and bootstrapped parametric likelihood ratio tests (BLRT); [76]. LMR and BLRT were applied to test whether a solution with *k + 1* groups fits the data significantly better than the solution with *k* groups. Further, relative entropy should be at least 0.8 as with a value of 1.0 indicating perfect classification [77]. However, there is no binding criterion to decide the number of trajectory classes. Instead, a variety of factors like theoretical considerations, interpretability or replicability among others should be considered [73].

Second, repeated measures analysis of variances with time as within-subjects factor and group membership as between-subjects factor was performed for general psychopathology for the three measurement points. In addition, survival analyses were run to examine the courses of lifetime diagnoses of depression and anxiety disorders over the ten year period. Further, we conducted logistic regression analyses to examine odds ratios (OR) for the T_2_ diagnoses of current depressive and anxiety disorders depending on developmental group and controlled for depression and anxiety symptoms at T_0_ (as measured with the YSR).

Third, data from EMA were aggregated into a mean experience score for valence, arousal and each specific emotion. Further, exploratory factor analysis with oblimin rotation was conducted with the specific emotion scores. In addition, mean squared successive differences (MSSD) within a day were calculated as a marker for emotional instability for a detailed description of the MSSD see [78],[79]. MSSDs were averaged over the assessment days for each participant and weighted by the emotion level, as there is evidence that absolute level and affect dynamics are interrelated [41]. Finally, a multivariate analysis of variances (MANOVA) was performed to identify group differences regarding valence, arousal, emotional factor values and emotional instability.

## Results

Descriptive statistics regarding demographic variables as well as neuroticism, psychopathology and everyday emotional experience can be seen in Table 2.

**Table 2:** **Descriptive statistics regarding demographics, neuroticism, psychopathology, prevalences of depression and anxiety disorders and emotional experience (n = 266)**

	%	n
**T**_**2**_ **demographics**		
education		
university degree	17.6	47
A-Levels	41.0	109
secondary school diploma	36.5	97
others	4.9	13
living in partnership	64.3	171
having children	12.8	34
**T**_**2**_ **current diagnosis**		
depression	9.8	26
anxiety	4.5	12
	M	SD
**Neuroticism**		
T_0_	2.26	(0.69)
T_1_	2.77	(0.58)
T_2_	2.55	(0.64)
**General psychopathology**		
T_0_	1.42	(0.25)
T_1_	1.39	(0.35)
T_2_	1.32	(0.40)
**T**_**2**_ **everyday emotional experience** ^**a**^		
valence^b^	1.89	(0.96)
arousal^c^	1.81	(0.87)

*Notes*. ^a^n = 222 due to missing values in ecological momentary assessment; ^b^ scaled from feeling good (0) to feeling bad (6); ^c^scaled from being relaxed (0) to being tense (6).

Using LCGA our models converged and fit indices for different class solutions can be seen in Table 3. In GMM, a non-significant negative residual variance (estimate -0.151, p = 0.07) of the slope factor occurred in the two group solution. This pattern did not change after modification of starting values and thus may indicate that there is no substantial variance of the slope factor within groups. Thus, for further examination we decided to go with the LCGA in which the variance of the slope factor is fixed to zero as this seemed to be a more proper model. In LCGA, differences in BIC were not wide, but it was the lowest for a three group solution (see Table 3). However, in this model entropy was slightly lower than 0.8 and the LMRT did not reach significance, indicating deficits in classification. Further, in this solution one class consisted of less than 10% of our sample which limits our confidence regarding the replicability of these results. As entropy was good in the two-group solution and LMR as well as BLRT were also significant in this model, we chose two trajectory classes for further analyses^a^. Estimated path coefficients for the model with two classes can be seen in Figure 1.

**Table 3:** **Fit indices for latent class growth analysis with neuroticism values at the three measurement points**

Number of groups	BIC^a^	Entropy	Lo-Mendell-Rubin likelihood ratio test	Bootstrapped parametric likelihood ratio test
			p^b^	p^b^
2	1430.303	0.833	.0005	.0000
3	1413.441	0.750	.1119	.0000
4	1417.614	0.687	.0655	.0000

*Notes*. ^a^Bayesian Information Criterion; ^b^testing if a model with *k* groups fits the data better than the model with *k-1* groups; group sizes: 2 class solution: n_1_ = 205, n_2_ = 61; 3 class solution: n_1_ = 162, n_2_ = 23, n_3_ = 81; 4 class solution: n_1_ = 48, n_2_ = 113, n_3_ = 98, n_4_ = 7.

**Figure 2:**
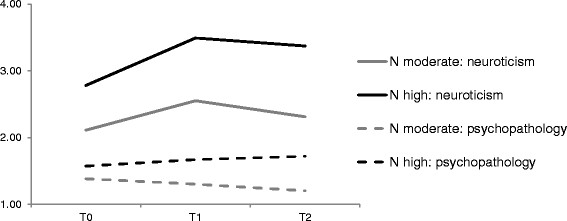
**Neuroticism and psychopathology course over the three measurement points for the two trajectory groups.** Legend: N_(N__moderate)_ = 205; N_(N high)_ = 61.

In both groups, neuroticism significantly changed from T_0_ to T_2_ with a peak at T_1_. However, the groups differed regarding absolute neuroticism levels. The majority of participants (77.1%; mean age T_0_ 14.75, SD = 2.20; mean age T_1_ 19.20, SD = 2.26; mean age T_2_ 24.73, SD = 2.31; 51.0% female) showed a pattern of moderate neuroticism values. Thus, this group was labelled “neuroticism (N) moderate” (mean intercept 2.084, SE = 0.362, p ≤ .001; mean slope 1.380, SE = 0.309, p ≤ .001). Individuals clustered into the second group (22.9%; mean age T_0_ 15.75, SD 2.04; mean age T_1_ 19.89, SD 1.76, mean age T_2_ 25.26, SD 1.96; 77.0% female) showed higher neuroticism levels (mean intercept 2.844, SE = 0.428, p ≤. 001) as well as slightly greater change over ten years (mean slope 1.666, SE = 0.377, p ≤. 001). This group was named “N high”. Neuroticism means for the trajectory groups are visualized in Figure 2.

Repeated measures analysis of variances revealed a significant main effect for trajectory group (F = 132.01, p ≤. 001, effect size partial eta squared (ƞ_P_^2^) = .33) as well as a significant interaction effect of group x time (F = 31.59, p ≤ .001, ƞ_P_^2^ = .11) on general psychopathology. The main effect for time did not yield significance (F = 0.21, p = .644, ƞ_P_^2^ = .001). Across the three assessments, the group “N high” showed higher psychopathological burden than individuals with stable moderate neuroticism values (see Figure 2). Further, in individuals with high neuroticism, psychopathology slightly increased in our ten year period whereas it decreased in the “N moderate” group.

**Figure 3:**
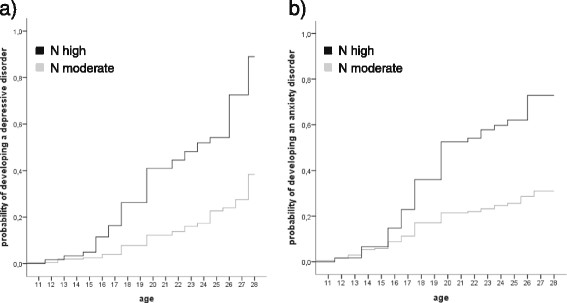
**Morbidity curves for depression (a) and anxiety disorders (b) according to neuroticism developmental group.** Legend: N = neuroticism; N_(N__moderate)_ = 205; N_(N high)_ = 61; the age 28 includes individuals who are 28 and older.

The results of survival analyses regarding the lifetime prevalences of depressive and anxiety disorders for groups can be seen in the morbidity curves in Figure 3. Curves differed significantly between groups (depression *χ*^2^ = 41.44, df = 1, p ≤ .001; anxiety *χ*^2^ = 28.84, df = 1, p ≤ .001) with elevated prevalences in the “N high” group. The gap between groups widened with increasing age. The estimated course shows that at an age of 28 or older nearly every person in the “N high” group suffered from depression or anxiety disorders at least once during their lives. Further, logistic regression analyses predicting current diagnoses at T_2_ revealed a 14-fold increased risk for depressive disorders in the “N high” compared to the “N moderate” group (β = 2.64, SE = 0.52, p ≤ .001, OR 14.00, confidence interval (CI) 5.08 - 38.34) controlled for internalizing symptoms at T_0_ (regression without trajectory group: β = 0.08, SE = 0.04, p = .035; regression with trajectory group: β = -0.02, SE = 0.05, p = .669). Regarding anxiety disorders a 7-fold risk was found for this high group (β = 1.92, SE = 0.74, p ≤. 01, OR 6.84, confidence interval (CI) 1.61 - 29.07; coefficients for the control variable internalizing symptoms at T0: without trajectory group: β = 0.17, SE = 0.05, p ≤ .001; regression with trajectory group: β = 0.11, SE = 0.05, p = .035).

**Figure 4:**
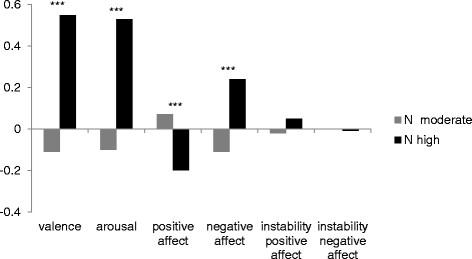
**Means of emotional experience in everyday life (EMA) according to neuroticism trajectory group.** Legend: N = neuroticism; N_(N__moderate)_ = 166; N_(N high)_ = 46; valence: higher values indicate feeling bad; arousal: higher values indicate being tense; instability is assessed with the MSSD; all values are z-standardized; ***p ≤ .001.

In the next step, we focused on information about emotional experience in everyday life as assessed by EMA. Completion rate was 88% with a mean of 18 answered calls. Data were analysed if at least 50% of calls were answered resulting in a sample of 208 individuals (“N moderate”: 162; “N high”: 46). A MANOVA revealed significant group differences in the global emotional indices valence (F = 17.54, p ≤. 001, ƞ_P_^2^ = .08) and arousal (F = 15.57, p ≤ .001, ƞ_P_^2^ = .07). Individuals with a neuroticism course on a moderate level felt better and were more relaxed during a two week period than individuals whose neuroticism values were higher (see Figure 4).

Further, exploratory factor analysis was conducted with ratings of specific emotions. Here, a two factor solution emerged. The first factor consisted of ratings for sadness, disgust, anxiety, anger, shame and boredom, and accounted for 57% of variance. This factor was labelled “negative affect”. Happiness and interest ratings constituted a second factor which accounted for 20% of variance and was named “positive affect”. As can also be seen in Figure 3, groups differed significantly in negative (F = 10.71, p ≤ .001, ƞ_P_^2^ = .05) and in positive affect (F = 10.39, p ≤ .001, ƞ_P_^2^ = .05). Individuals with high neuroticism values experienced more negative and less positive affect in everyday life than the “N moderate” group. According to the results of the factor analysis, the MSSD was calculated separately for positive and negative affect. However, groups did not differ regarding emotional instability, neither in positive (F = 0.17, p = .682, ƞ_P_^2^ = .001) nor in negative affect (F = 0.01, p = .929, ƞ_P_^2^ = .000).

## Discussion

In this study we longitudinally examined differential developmental courses of neuroticism from adolescence to young adulthood, and their association to psychopathology and emotional experience in a general population sample. In particular, various levels of psychopathology were assessed using self-reports, structured clinical interviews, and an ecological momentary assessment approach.

As hypothesized, neuroticism course was not uniform across individuals in our general population sample. Instead, over a period of ten years, two different forms of neuroticism development were revealed. In both groups, neuroticism was not stable, but changed from adolescence to young adulthood as indicated by the significant slope factors. Interestingly, the shape of the neuroticism course was similar in both groups with a peak around the age of 20. In contrast, courses differed regarding absolute neuroticism level. The majority of individuals showed neuroticism values on a moderate absolute level. However, there was also a group with higher absolute neuroticism levels. This higher pattern was associated with an elevated level of psychopathology from adolescence to young adulthood. Further, individuals who stood out from the masses by being more timid, nervous and emotionally unstable were at 14-fold increased risk for developing depressive and at 7-fold risk for anxiety disorders compared to persons with moderate neuroticism levels. These results are in line with studies associating lower levels of neuroticism with positive outcomes such as life satisfaction [80] or subjective well-being [81].

According to the maturity principle [13], a decrease of neuroticism values would have been expected in the majority of individuals. This pattern could not be observed in our data. Instead, neuroticism increased from 15 to 20 and decreased afterwards in both groups. This pattern might be explained by the model of Ormel et al. [82]. Here, the authors provide evidence that personality development bases on two factors: on the one hand, there is an individually fixed set point. On the other hand, there are experience-dependent alterations in personality. Hence, it can be assumed that in line with personality models some individuals do have a higher neuroticism set point than others [5]. At the same time, the age of 20 reflects an important developmental step associated with experiences that potentially lead to an increase in neuroticism values. It is easily imaginable that moving out, finding a job and perhaps starting an own family can fuel fears, worries and negative emotions. Perhaps, five years later individuals become more settled which is reflected in a decrease of neuroticism values back to the respective set point. This assumption is in line with findings showing a decrease in neuroticism between 20 and 40 [14]. In fact, lots of studies reporting decreases in neuroticism assess individuals older than 18 years [23],[83]. Hence, it seems plausible that hypothesized personality maturation is just about to start in our sample. Of course, this must be clarified in future studies which should also account for potentially different paces of such maturation processes.

In parallel to the neuroticism course, morbidity rates of anxiety and depressive disorders strongly rose from T_0_ to T_1_ but the increase slowed down from T_1_ to T_2,_ particularly in the “N high” group. This is in line with other studies showing increases in depressive symptoms starting in the ages between 12 and 14 [84],[85]. Hence, our data might suggest that in parallel with increasing neuroticism values from T_0_ to T_1_, depressive and anxiety symptoms reach the threshold of diagnoses at the age of 20, particularly in individuals with a higher absolute level of neuroticism. However, it could be argued, that these associations result from the conceptual overlap of neuroticism and psychopathology measures [86]. Yet, there is evidence suggesting that content overlap is not the main explanation for associations between neuroticism and depression/anxiety [9]. Instead, neuroticism seems to reflect more than depressive and anxious symptoms, as a general neuroticism factor including all of its facets is a better predictor for depression and anxiety than the disorder-specific subscales [9]. Further, general psychopathology measures also include externalizing symptomatology and thus are supposed to be sufficiently distinct from neuroticism. Nevertheless, it is possible that the strength of the association is a little overestimated. Hence, in line with Nicholls et al. [87], we decided not to exclude overlapping items but to include a variety of outcome assessment methods to account for potential conceptual overlap.

Further, it could be assumed that retrospective recall of symptoms and personality is biased by current psychopathology and mood [88],[89]. Therefore, we additionally used EMA to assess implications of neuroticism developmental groups. This method minimizes recall biases and other systematic distortions, as individuals spontaneously indicate their current emotional experience at multiple random assessment points. However, this method was only applicable at T_2,_ so recall biases at T_0_ and T_1_ cannot be ruled out.

Using EMA at T_2_, we found high neuroticism course from adolescence to adulthood to be associated with increased negative affect and arousal at the age of 25. In addition, levels of positive affect were reduced. This is of particular importance, as there is evidence that negative emotions in everyday life are associated with various adverse outcomes, such as an increased vulnerability for depression [90], smoking relapse [91], or binge eating [92], for instance. Further, Wichers et al. [93] found positive emotions in everyday life to buffer the disadvantageous effects of stress on depression development. Hence, the high neuroticism group is affected in two ways: first, by its increased negative affectivity, and second, through the lack of possibly protecting positive emotions. In sum, it can be assumed that alterations in emotional experience constitute a mechanism relating neuroticism development to psychopathology. This idea needs clarification in future research.

Interestingly, no group differences emerged regarding emotional instability. This is in contrast to other studies reporting significant associations between neuroticism and affect instability [41],[46],[94]. However, these studies did not test the influence of longitudinal neuroticism courses on emotional instability, but assessed or averaged concurrent neuroticism levels instead. Another methodological explanation for these inconsistent findings is provided in a recent study of Koval, Pe, Meers, and Kuppens [31]. They argue that overlap in conceptualizations (variability, instability, inertia) and measures (SD, MSSD, autocorrelation) of affect dynamics account for inconsistencies in results (in their case regarding depression). Thus, it would be interesting to test whether neuroticism courses differentially influence diverse measures of affect dynamics in future studies.

Our results have to be interpreted in the light of several limitations. Although data were collected longitudinally, causal statements cannot be made. Whereas in our argumentation the developmental course of neuroticism is interpreted as risk factor for different negative outcomes, it is also plausible that differences in mental stress influence personality (for review see [95]). For instance, evidence is inconsistent regarding depression: whereas the *vulnerability hypothesis* states that personality constitutes a risk factor for depressive disorders [96],[97] the *scar hypothesis* arguments that an episode of depression leads to alterations in personality [98]. In our study we found evidence for the vulnerability hypothesis, as a neuroticism course with high absolute levels led to an increased risk of depression in adulthood. However, it would be promising to examine whether previous depressive episodes influenced personality development in a future study.

Further, it is also imaginable that third factors like a family history of mental illness [99], treatment experiences [100], significant life events [101] or traumas [102] influence the associations between neuroticism course and mental health. Hence, such mechanisms should be considered in further research. In addition, the concurrent assessment of personality and psychopathology might lead to mood-state distortions [103]. Hence, for the future it might be promising to assess personality and psychopathology at different time points controlling for current mood-state.

Moreover, in our EMA-design we did not capture the context in which emotions were experienced. However, there is increasing evidence emphasizing the importance of context-specific information on emotions [104],[105]. Therefore including a few questions on the activities, stressors and interactions partners in everyday situations might help to get a more detailed insight in the emotionality of individuals at risk for depression and anxiety disorders. However, EMA research is still at its very beginning and our results give a first idea of the association between trait affectivity and affective experience in everyday life.

Further, in this study we focused on the personality trait neuroticism, as this trait is frequently examined in the context of internalizing psychopathology [106],[107]. Of course, the development of other traits such as extraversion or impulsivity would also be interesting as these traits are discussed as risk factors of mental disorders, too [8],[108]. In addition, future research should examine the association between the development of trait combinations in terms of personality profiles and psychopathology.

Methodologically, varying assessment methods for neuroticism and general psychopathology were used at the different measurement points. This adaption was inevitable due to age-specific application of the questionnaires. However, we included age as a covariate in our models to account for possible assessment effects. Still, method-specific biases cannot completely be ruled out. Thus, overcoming measurement problems is a major challenge for future research on personality development in the transition from adolescence to adulthood.

Nevertheless, to the best of our knowledge, this is the first study differentiating courses of neuroticism development in this phase of life and longitudinally linking them to different forms and severities of psychological impairment. Therefore, a multimethod approach with self-reports, interview data and ecological momentary assessment was used. Further, our data were collected in both sexes in the general population instead of patient samples or college students, thus enhancing the generalizability of our findings.

## Conclusions

This study highlights that neuroticism changes in the transition from adolescence to young adulthood. However, personality maturation as indicated by a decrease of neuroticism could not be observed. Instead, neuroticism peaked at the age of 20. Interestingly, this form of development was similar across individuals. However, the absolute neuroticism level strongly differed between two groups. Over 20% of our participants showed elevated neuroticism levels over all assessments which were associated with adverse outcomes such as negatively toned emotional experience, increased general psychopathology over ten years, and a heightened risk to suffer from depression and anxiety disorders in young adulthood. Thus, these high-risk persons need to be identified early to be able to provide individually suited interventions to support continuous personality maturation. At the same time, the assessment of possible negative outcomes needs to be refined in order to detect specific patterns increasing the risk for mental disorders, such as increased experience of negative emotions in everyday life.

## Endnote

^a^We also tested a model including a dummy-coded control variable, indicating whether J-TCI or NEO was used to assess neuroticism at T_0_ instead of age (results not shown). Here, a similar 2-class-solution was obtained and further results were comparable. As it was age-dependent whether the J-TCI or the NEO was used, age and the control variable were highly interrelated (r = 0.862, p ≤ .001). Hence, we decided to display the age-controlled model only.

## Authors’ contributions

MA made substantial contributions to the conception and design of the study, acquisition, statistical analyses and interpretation of the data (particularly EMA), and wrote the first draft of the manuscript. MS made substantial contributions to the conception and design of the study as well as to statistical analyses and revised the manuscript critically for important intellectual content. IU made substantial contributions to the conception and design of the study, and revised the manuscript critically for important intellectual content. KA made substantial contributions to the conception and design of the study, had the lead in diagnostics and revised the manuscript critically for important intellectual content. ER made substantial contributions to the conception and design of the study, and revised the manuscript critically for important intellectual content. SW made substantial contributions to the acquisition of data, and revised the manuscript critically for important intellectual content. HJG made substantial contributions to the acquisition of data, and revised the manuscript critically for important intellectual content. SL revised the manuscript critically for important intellectual content. SB made substantial contributions to the conception and design of the study, the analysis and interpretation of the data, and revised the manuscript critically for important intellectual content. All authors contributed to and have approved the final manuscript.

## Authors’ original submitted files for images

Below are the links to the authors’ original submitted files for images. Authors’ original file for figure 1 Authors’ original file for figure 2 Authors’ original file for figure 3 Authors’ original file for figure 4

## Abbreviations

BIC: Bayesian information criterion
BLRT: Bootstrapped parametric likelihood ratio tests
EMA: Ecological momentary assessment
HA: Harm avoidance
LCGA: Latent class growth analysis
LMR: Lo-Mendell-Rubin-test
MSSD: Mean squared successive differences
N: Neuroticism
OR: Odds ratio
SHIP: Study of health in Pomerania
