# Supplementary figures and images for: Neuroticism developmental courses - implications for depression, anxiety and everyday emotional experience; a prospective study from adolescence to young adulthood
Source: BMC Psychiatry. 2014 Aug 6;14:210. doi: 10.1186/s12888-014-0210-2 (PMC4158099; doi:10.1186/s12888-014-0210-2)

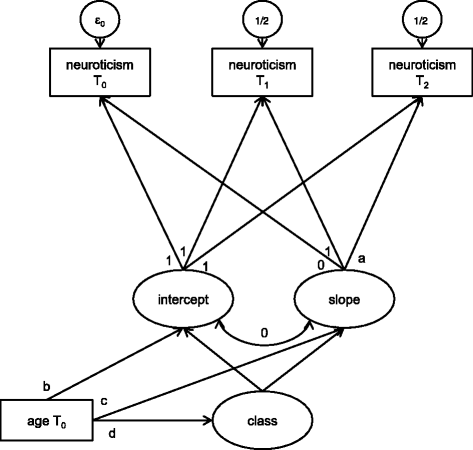

Supplement: Supplementary file 1 — Authors’ original file for figure 1 [file 12888_2014_210_MOESM1_ESM.gif]

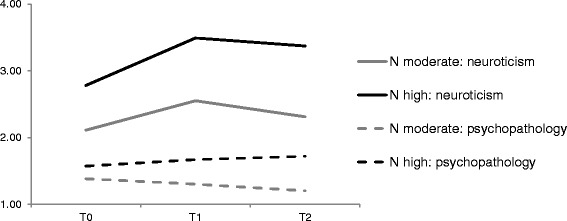

Supplement: Supplementary file 2 — Authors’ original file for figure 2 [file 12888_2014_210_MOESM2_ESM.gif]

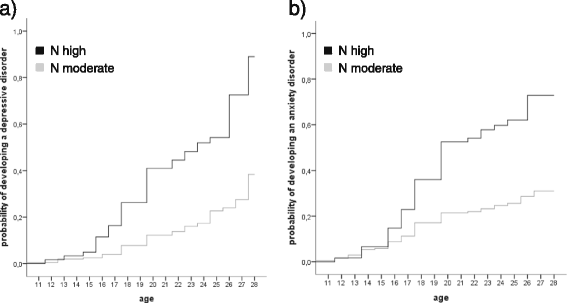

Supplement: Supplementary file 3 — Authors’ original file for figure 3 [file 12888_2014_210_MOESM3_ESM.gif]

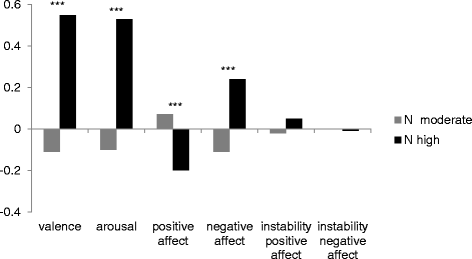

Supplement: Supplementary file 4 — Authors’ original file for figure 4 [file 12888_2014_210_MOESM4_ESM.gif]
